# Supersaturated Oxygen Therapy for Coronary Microvascular Dysfunction in Patients With ST-Segment Elevation Myocardial Infarction: The IC-HOT-MICRO Pilot Study

**DOI:** 10.1016/j.jscai.2026.104269

**Published:** 2026-04-16

**Authors:** Benoit Caullery, Ikram El Marzouki, Stephanie Marliere, Estelle Vautrin, Nicolas Piliero, Helene Bouvaist, Gerald Vanzetto, Jean Luc Bosson, Gilles Barone-Rochette

**Affiliations:** a Department of Cardiology, University Hospital, Grenoble, France; b University Grenoble Alpes, INSERM, CHU Grenoble Alpes, LRB, Grenoble, France; c French Clinical Research Infrastructure Network, Paris, France; d Public Health Department, University Grenoble Alpes, Clinical Investigation Center-Technological Innovation, INSERM CIC1406, CHU Grenoble Alpes, Grenoble, France; e University Grenoble Alpes, Méthodologie de l'information en Santé, Biostatistiques, Recherche Clinique et Innovation Technologique, Pôle Santé Publique, CHU Grenoble Alpes, Grenoble, France

**Keywords:** acute coronary syndrome, coronary microvascular dysfunction, supersaturated oxygen therapy

## Abstract

**Background:**

Supersaturated oxygen (SSO_2_) therapy is a new therapy that reduces infarct size and improves myocardial remodeling after successful revascularization by percutaneous coronary intervention (PCI). An action mode via the improvement of coronary microvascular dysfunction (CMD) is suggested; however, the device requires a femoral puncture and keeping the patient in the interventional room for 60 minutes. Thus, the development of stratified medicine via CMD severity could be of interest. This study sought to investigate the action of SSO_2_ therapy on CMD in patients with anterior ST-segment elevation myocardial infarction (STEMI).

**Methods:**

In a prospective pilot study, consecutive patients with acute anterior STEMI undergoing primary PCI were enrolled. After successful PCI of a proximal lesion or middle left anterior descending artery, SSO_2_ therapy was performed. Blood was drawn from a femoral artery sheath (5F catheter), hyper-oxygenated by SSO_2_ therapy, and delivered to the origin of the left anterior descending artery a via a dedicated catheter by radial artery (6F catheter). Improvement in CMD was assessed by comparing the angiography-derived index of microcirculation resistance (Angio-IMR) before and after 60 minutes of SSO_2_ therapy measured on conventional angiographic images. ΔAngio-IMR was calculated as Angio-IMR before SSO_2_ – angio-IMR after SSO_2_.

**Results:**

Twelve patients (all men; mean age, 64 [59-69] years) successfully completed the procedure. No complications were noted during the SSO_2_ procedure. Median Angio-IMR before SSO_2_ was 44.5 (32.5-52.5) and median Angio-IMR after SSO_2_ was 23 (20-30.5); *P* = .003. ΔAngio-IMR was statistically different between patients with or without Angio-IMR >40 at baseline (25 [16.2-29.7.5] vs 4.5 [1-11]; *P* = .004).

**Conclusions:**

In patients with anterior STEMI, SSO_2_ improves CMD measured by angio-IMR. Only patients with high Angio-IMR at baseline (>40) presented a significant decrease. These results need to be confirmed with more data, and thus, Angio-IMR could help to develop stratified medicine to use SSO_2_ in anterior STEMI patients with severe CMD.

## Introduction

Myocardial infarction (MI) remains one of the leading causes of mortality worldwide.^1^ Despite advances in its management,^2^ morbidity and mortality have plateaued since 2010, particularly among patients with ST-segment elevation myocardial infarction (STEMI).^3^ The 1-year mortality rate is approximately 7.5%,^4^ and the risk of developing heart failure ranges between 20% and 30%.^5^ These figures highlight the urgent need for new therapeutic strategies, especially those targeting the coronary microcirculation, where coronary microvascular dysfunction (CMD) has emerged as a powerful prognostic marker ^6^^,^^7^ Despite TIMI 3 epicardial flow after primary percutaneous coronary intervention (PCI), impaired myocardial tissue perfusion persists in ∼30% to 40% of STEMI patients—a spectrum encompassing microvascular obstruction and CMD.^8^ CMD is a powerful, independent predictor of adverse outcomes; meta-analyses report ∼3.4-fold higher risk of major adverse cardiovascular events in patients with severe CMD after STEMI.^9^ Given a post-PCI CMD prevalence of ∼30% to 40% and a major adverse cardiovascular event hazard ratio around 3.4, a simple population-attributable risk calculation suggests that roughly 40% to 50% of the residual post-MI risk could relate to CMD, acknowledging heterogeneity in definitions, end points, and time horizons.^8^ This helps explain the plateau in outcomes despite optimized epicardial reperfusion and guideline-directed therapy, and it motivates trials targeting the microcirculation as a complementary therapeutic axis.

In the catheterization laboratory, visual assessment tools such as the TIMI frame count do not reliably evaluate CMD.^7^ The index of microcirculatory resistance (IMR),^10^ which can be measured invasively during coronary angiography, has demonstrated superior prognostic value^9^ and holds promise as a theragnostic biomarker. Stratified medicine, which tailors treatment to specific patient subgroups, presents a compelling opportunity for the clinical application of IMR.^8^ Additionally, IMR can be calculated from coronary angiography using software (Angio-IMR), offering a cost effective and safer alternative for patients.^11^

Supersaturated oxygen (SSO_2_) therapy, approved by the FDA, involves the infusion of highly oxygenated blood into the coronary artery following successful revascularization.^12^ This therapy has been shown to reduce infarct size by a median of 26%.^13^ Clinical trials, including AMIHOT I, II, and IC-HOT, have confirmed its safety and efficacy.^13^, ^14^, ^15^, ^16^, ^17^ The beneficial effect of SSO_2_ is thought to result from improvements in CMD,^18^ although this has yet to be demonstrated in humans.

Because Angio-IMR has been validated against invasive IMR,^11^ a rapid decrease in Angio-IMR following SSO_2_ therapy could serve as evidence of its effect on CMD. We hypothesize that SSO_2_ therapy will improve CMD, as reflected by a measurable reduction in Angio-IMR.

## Materials and methods

### Study design and population

The study was a prospective study with 1 cohort and no comparison group. We included only patients who had suffered an MI caused by occlusion of the left anterior descending artery (LAD) (only patients with anterior MI related to proximal or proximal-to-mid LAD occlusion were included, in line with previous studies, and to ensure a sufficiently large myocardial area at risk). Patients enrolled in this study were consecutive individuals aged over 18 years admitted for STEMI within 6 hours of symptom onset. Eligible ECG criteria include ST-segment elevation ≥1 mm in ≥2 contiguous precordial leads (V1-V4) or new-onset left bundle branch block. All participants must have undergone successful PCI for a proximal or mid LAD lesion, using commercially available drug-eluting stents (DES) and achieving TIMI flow grade 2 or 3. An arterial partial pressure of oxygen (PaO_2_) ≥10.7 kPa (80 mm Hg), whether with or without supplemental oxygen, is also required. Exclusion criteria included were: prior coronary artery bypass grafting or MI, history of PCI of the LAD, or planned PCI of the LAD within 30 days, mechanical complications of STEMI, cardiogenic shock, or use of an intraaortic balloon pump, severe valvular disease, heart failure, pericardial disease, or nonischemic cardiomyopathy, documented left ventricular ejection fraction (LVEF) <40%, use of fibrinolytic therapy, contraindications to anticoagulation, including creatinine clearance <30 mL/min/1.73 m^2^, hemoglobin <10 g/dL, recent gastrointestinal or urogenital bleeding (within 2 months), or any major surgical procedure, including planned aortic valve replacement, within the past 6 weeks. A total of 15 patients were enrolled. Angio-IMR measurements before and after SSO_2_ were available in 12 patients, who, therefore, constituted the final analysis population.

### PCI procedure

Procedural details and medication protocol were: Use of glycoprotein IIb/IIIa inhibitors, balloon predilatation, and manual thrombus aspiration, which was left to the discretion of the interventional cardiologist. Unfractionated heparin was administered for procedural anticoagulation. All patients received 250 mg of aspirin and a loading dose of clopidogrel, prasugrel, or ticagrelor prior to coronary angiography. PCI was performed following standard practice using DES, as clinically indicated. At discharge, patients were prescribed aspirin (75 mg/d) indefinitely, alongside dual antiplatelet therapy for 1 year. In the absence of contraindications, high-intensity statins, β-blockers, and ACE inhibitors or angiotensin receptor blockers were also initiated, in accordance with European guidelines.^19^

### SSO_2_ therapy

Supersaturated oxygen therapy was administered immediately after successful PCI. Oxygenated blood was drawn from the femoral arterial sheath and circulated through an extracorporeal oxygenation circuit, incorporating a roller pump and a polycarbonate oxygenator chamber (TherOx), achieving a PaO_2_ between 760 and 1000 mm Hg, in line with previously reported protocols.^13^ To deliver the hyperoxemic solution, the left main coronary artery was selectively cannulated using a 5F soft-tip Judkins left diagnostic catheter (IMPULSE, Boston Scientific). The SSO_2_-enriched blood was infused at a steady flow rate of 100 mL/min over a 60-minute period, entirely within the cardiac catheterization suite. Systemic PaO_2_ levels were verified before the start of the infusion, and nasal oxygen supplementation was titrated as necessary to maintain PaO_2_ ≥80 mm Hg. Unfractionated heparin was administered during the procedure. ACT was monitored regularly (approximately every 20 minutes) and maintained above 250 seconds before and during the SSO_2_ infusion. If the ACT dropped below this threshold, a supplemental 3000 IU IV bolus of heparin was administered and the ACT was rechecked to ensure restoration of the target level. It should be noted that in our study, 100% of the radial approach was combined with a femoral approach. Femoral ultrasound-guided puncture was performed after successful PCI. This strategy is in line with international recommendations favoring the radial access for the management of STEMI.^19^^,^^20^

### Angiography-derived index of microcirculatory resistance (Angio-IMR)

In this study, microvascular resistance was assessed exclusively using Angio-IMR derived from coronary angiography. No wire-based thermodilution IMR measurements were performed. Following PCI, intracoronary nitroglycerin was systematically administered (when systolic blood pressure permitted), and baseline Angio-IMR was measured. After the 60-minute SSO_2_ infusion, intracoronary nitroglycerin was administered again, and Angio-IMR was reassessed. Coronary angiography was performed using standard high-frame-rate contrast injection protocols (3 cardiac cycles, 15 frames per second). For analysis, the most appropriate end-diastolic frame—offering optimal vessel opacification and minimal overlap—was selected in both projections. Angio-IMR was computed using QAngio XA 3D software (QFR, version 2.1 Research Edition; Medis Medical Imaging), as described in previous work.^21^ All evaluations were performed by a single reader (G.B.-R.), blinded to the clinical data. The stepwise process included: (1) selection of 2 angiographic runs with angular separation >25°, (2) identification of end-diastolic frames with optimal contrast filling, (3) annotation of anatomical landmarks for 3D spatial correction, (4) manual delineation of the vessel lumen and setting of proximal and distal reference points, (5) manual TIMI frame counting and insertion of resting aortic pressure (Pa), and (6) reconstruction of the 3D vessel geometry and reference surface with concurrent computation of QFR and Angio-IMR values. The formula applied for Angio-IMR calculation was as follows:

$$\text{Angio}-\text{IMR}=\left(e-{\text{Pa}}_{h}\text{yp}\right)\times \text{QFR}\times \left(\text{vessel}\;\text{length}/V_{h}\text{yp}\right)$$

where Pa = mean resting aortic pressure, e-Pa_h_yp = estimated hyperemic Pa, derived by applying a correction factor to resting Pa: e-Pa_h_yp = Pa_rest – (0.1 × Pa_rest),^22^ vessel length was defined as the segment used for QFR computation, V_h_yp = modeled hyperemic flow velocity, estimated via a quadratic model based on the resting TIMI frame count.^23^ Vessel length was the centerline length of the 3D-reconstructed LAD segment used for QFR computation (between user-defined proximal and distal reference points). Resting TIMI frame count was determined manually on the same angiographic run at rest (no adenosine) using standard methodology: the start frame was defined as the first frame with full opacification of the proximal LAD, and the end frame as the frame when contrast reached a predefined distal landmark (eg, distal bifurcation).

A threshold value of Angio-IMR >40 was considered indicative of CMD.^24^^,^^25^ All image processing was blinded to clinical outcomes, and analyses were performed using the certified version of the software used during the training phase by the investigators (G.B.-R., B.C.). The Central Illustration depicts the protocol.

**Central Illustration:**
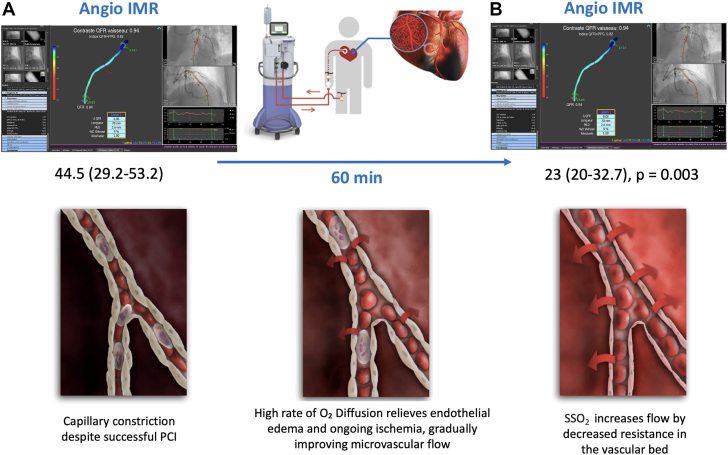
**Protocol and microcirculatory effect of supersaturated oxygen (SSO_2_) therapy in anterior ST-segment elevation myocardial infarction.** (A) Pre-SSO_2_ Angio-IMR assessment obtained after primary percutaneous coronary intervention (PCI), characterizing the degree of coronary microvascular dysfunction. (B) Post-SSO_2_ reassessment following intracoronary SSO_2_ infusion, showing the resulting improvement in microvascular resistance. This figure illustrates the overall procedural workflow and the measurable impact of SSO_2_ on coronary microcirculation.

### Echocardiographic analysis

All participants underwent a comprehensive transthoracic echocardiographic examination using a commercially available ultrasound system (Vivid E95; GE Medical Systems) equipped with a 2.5 MHz phased-array transducer. Image acquisition followed standard protocols, and data were analyzed offline using dedicated software (EchoPAC PC, version 203; GE Medical Systems). A baseline echocardiogram was performed within 24 hours of hospital admission, and a follow-up assessment was conducted 3 months after the index MI. LVEF and left ventricular (LV) volumes were measured using the biplane modified Simpson’s method. Adverse LV remodeling was defined as an increase of ≥20% in the LV end-diastolic volume between baseline and the 3-month follow-up. Importantly, the cardiologists conducting the analyses were blinded to both the stent type and the IMR values.

### Cardiac magnetic resonance imaging

Cardiac magnetic resonance imaging (CMR) was conducted using both 1.5-T and 3-T scanners (Magnetom Aera and Skyra; Siemens Healthineers AG). A series of 10 to 12 contiguous short-axis cine slices encompassing the entire left ventricle was obtained. Additionally, 2-, 3-, and 4-chamber long-axis cine views were acquired using a steady-state free precession sequence to assess global myocardial function and mass. Approximately 8 to 10 minutes after administering a gadolinium-based contrast agent (0.2 mmol/kg), the same short- and long-axis imaging planes were repeated using a 2D or 3D inversion-recovery sequence, optimized for detecting late gadolinium enhancement.

Image postprocessing was carried out by a single blinded reader (G.B.-R.) with over 10 years of experience and EACVI level 3 certification, who was unaware of the patients’ coronary status. Ventricular volumes and myocardial mass were calculated using QMass software (Medis). Infarct size was quantified using a signal intensity threshold set at >6 standard deviations (SD) above remote myocardium. Final infarct size was expressed as a percentage of the total myocardial mass.

### Cardiopulmonary exercise testing

In this study, cardiopulmonary exercise testing was performed on a cycle ergometer (Ergometrics 900; Ergoline) to evaluate peak oxygen uptake ($\dot{V}O_{2}$peak). Throughout the test, heart rate, blood pressure, 12-lead ECG, and breath-by-breath gas exchange were continuously recorded.

Participants began with a 10-minute standardized warm-up at low intensity (20-30 W). The test was then initiated at a starting workload of 30 to 50 W, personalized according to the individual’s fitness level. Workload was subsequently increased incrementally by 10 to 15 W per minute, allowing for sufficient physiological adaptation at each stage.

The $\dot{V}O_{2}$peak was defined based on the presence of a plateau in oxygen consumption despite further increases in workload, alongside a respiratory exchange ratio ≥1.05. Heart rate was monitored continuously, and the test was terminated either at the point of volitional exhaustion or when preestablished maximal effort criteria were met.

### End points

The primary end point of the study was the difference between Angio-IMR after 60 minutes of SSO_2_ and at baseline. The secondary end points were descriptive only with 30-day composite rate of net clinical adverse events (death, reinfarction, target vessel revascularization, stent thrombosis, severe heart failure, or major/minor TIMI bleeding), measurement of exercise performance by cardiopulmonary exercise testing, reversal of cardiac remodeling by echocardiography, and measurement of infarct size by CMR.

### Statistical analysis

The number of patients to include is based on the primary criterion of improvement in CMD assessed by Angio-IMR. Based on previously published data, the average Angio-IMR has been estimated at 60 units for a sample of patients with STEMI and CMD. A sample of 10 patients was necessary to demonstrate a 50% improvement in Angio-IMR after SSO_2_ treatment, with a power of 90%, using a 2-tailed paired *t* test, considering a common SD of 20 units and an alpha risk of 5%, and accounting for some refusals to participate during the study.^11^ To ensure at least 10 analyzable cases, we, therefore, planned to enroll 15 patients to compensate for potential missing or nonassessable data.

The primary end point was analyzed using a nonparametric Wilcoxon signed-rank test to account for the “before/after” changes resulting from SSO_2_ therapy on CMD. Furthermore, to assess the reproducibility of the measures using Angio-IMR, 10 randomly selected patients with Angio-IMR analysis were evaluated twice by the same observer for intraobserver variability, and by 2 different observers for interobserver variability. Variability was quantified computing the intraclass correlation coefficient (ICC). Interobserver reliability for measurement of the Angio-IMR was assessed by using a 2-way random single-measure ICC analysis. Intraobserver and intrasubject reliability was assessed by using a 1-way random 2-measure ICC analysis. Reproducibility analyses were performed on 10 patients randomly selected from the study cohort itself. A 2-sided *P* value ≤.05 was considered statistically significant. All secondary criteria were analyzed descriptively. Descriptive statistics were presented using medians (interquartile ranges) and means (SD) for quantitative variables, and counts and percentages for qualitative variables. SPSS V26.0 statistical software system (SPSS Inc) was used for calculations and GraphPad Prism version 8.0 (GraphPad Software) for figures.

This study received institutional review board approval in compliance with current regulations. The trial sponsor was our University Hospital, and the study was conducted in accordance with the Declaration of Helsinki. The registry identifier for this trial, called “IC-HOT-MICRO,” on ClinicalTrials.gov is NCT05790876.^26^

## Results

Fifteen patients were initially included in the study. Among them, 12 patients had both baseline and post-SSO_2_ Angio-IMR measurements and were included in the primary analysis (mean age, 64 [59-69] years). Fifteen patients were initially enrolled. Three were subsequently excluded from the final analysis. In 2 patients, the PaO_2_ was actually <10.7 kPa (80 mm Hg) at inclusion, but this was misinterpreted at the time of screening, leading to initiation of SSO_2_ therapy; as these patients did not meet the predefined physiological inclusion criterion, they were excluded from analysis. In 1 additional patient, SSO_2_ infusion was discontinued after approximately 20 minutes because of a >90-second interruption of flow during ACT sampling (the SSO_2_ system detected a circuit pressure anomaly and shut down safely); according to the device operating protocol, resuming infusion required cartridge replacement and system repriming, which was not performed during the procedure, and therefore, this patient was also excluded. No complications were noted during implantation. Only 1 patient underwent revascularization of the LAD with 2 DES; all others underwent revascularization with a single DES.

**Figure 1:**
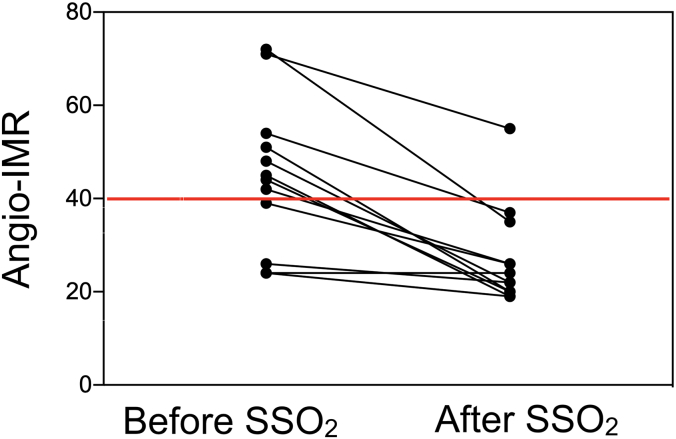
**Evolution of Angio-IMR before and after supersaturated oxygen (SSO_2_) therapy.** Individual patient values are shown before and after intracoronary SSO_2_ infusion, demonstrating the reduction in microvascular resistance associated with treatment (median Angio-IMR before SSO_2_ was 44.5 [32.5-52.5], and median Angio-IMR after SSO_2_ was 23 [20-30.5], *P* = .003).

Across the cohort, Angio-IMR decreased after SSO_2_. The reduction was present overall and was more pronounced in patients with elevated baseline Angio-IMR (>40). The clinical characteristics are presented in Table 1. Median Angio-IMR before SSO_2_ was 44.5 (32.5-52.5) and median Angio-IMR after SSO_2_ was 23 (20-30.5); *P* = .003. ΔAngio-IMR was statistically different between patients with or without Angio-IMR >40 at baseline (25 [16.2-29.7.5] vs 4.5 [1-11]; *P* = .004). Figure 1 shows Angio-IMR evolution before/after SSO_2_ therapy. No patient experienced the primary end point, a composite of net clinical adverse events (death, reinfarction, target vessel revascularization, stent thrombosis, severe heart failure, or major/minor TIMI bleeding) at 30 days.

**Table 1:** Clinical and STEMI characteristics.

Variable	Total population (N = 12)	IMR ≤40 (n = 4)	IMR >40 (n = 8)	*P* value
Male sex	100%	100%	100%	—
Age, y	64 (59-69)	66 (63-70)	62 (56-68)	.306
Body mass index, kg/m^2^	25.3 (24.1-26.9)	25.2 (24.4-26.4)	25.2 (23.8-26.6)	.808
Heart rate, bpm	74 (57-80)	76 (67-81)	74 (58-80)	.864
Hypertension	33.3%	25%	37.5%	>.99
Diabetes	8.3%	0%	12.5%	>.99
Smoking	8.3%	0%	12.5%	>.99
Dyslipidemia	50%	50%	50%	>.99
Coronary heredity	25%	0%	37.5%	.491
Thrombectomy	16.7%	0%	25%	.515
Predilation	50%	100%	25%	.061
Postdilation	16.7%	25%	12.5%	>.99
Symptom-to-door time, min	158 (131-209)	181 (145-210)	155 (132-193)	.68
SBP before SSO_2_ therapy, mm Hg	103 (93-126)	92 (89-96)	120 (99-134)	.109
DBP before SSO_2_ therapy, mm Hg	67 (58-74)	62 (56-66)	69 (63-77)	.233
SBP after SSO_2_ therapy, mm Hg	117 (98-148)	104 (95-108)	129 (112-159)	.106
DBP after SSO_2_ therapy, mm Hg	71 (65-84)	68 (60-75)	74 (66-87)	.283
Angio-IMR before SSO_2_ therapy	44.5 (32.5-52.5)	25.0 (24.0-29.2)	49.5 (44.8-58.2)	.008
Angio-IMR after SSO_2_ therapy	23 (20-30.5)	23.0 (21.2-24.5)	24.0 (20.0-35.5)	.608
LVEF initial by TTE, %	45 (43-47)	45 (45-46)	44 (39-48)	.604
eGFR, mL/min	91 (74-97)	87 (81-93)	91 (66-98)	>.99
Peak CPK, UI/L	3074 (2003-3712)	2723 (2079-3103)	3122 (2280-4594)	.461
3-month follow-up				
LVEF by TTE, %	54.0 (50.8-55.5)	56.0 (54.0-60.8)	53.0 (47.5-55.0)	.199
LVEF by CMR, %	46.0 (38.0-52.8)	55.0 (47.8-59.2)	41.0 (37.5-49.5)	.147
Cardiac mass by CMR, g/m^2^	54.7 (48.5-59.5)	48.2 (40.0-56.4)	56.6 (51.6-59.3)	.497
RVEF by CMR, %	59.0 (52.0-61.5)	61.0 (56.0-62.5)	57.0 (51.8-60.5)	.539
LGE extent, % of total LV mass	27.9 (19.5-33.8)	29.4 (28.7-29.8)	22.1 (16.2-43.8)	.551
$\dot{V}O_{2}$peak, mL/kg/min	22.4 (18.9-24.0)	20.5 (18.2-22.8)	22.7 (19.2-25.4)	.344
Adverse remodeling, %	25%	50%	12.5%	.236

Values are mean ± SD, median (IQR), or n (%).

Angio-IMR, angiography-derived index of microcirculatory resistance; CMR, cardiac magnetic resonance imaging; CPK, creatine phosphokinase; DBP, diastolic blood pressure; eGFR, estimated glomerular filtration rate; IMR, index of microcirculatory resistance; LGE, late gadolinium enhancement; LV, left ventricular; LVEF, left ventricular ejection fraction; RVEF, right ventricular ejection fraction; SBP, systolic blood pressure; SSO_2_, supersaturated oxygen; STEMI, ST-segment elevation myocardial infarction; TTE, transthoracic echocardiography.

At 3 months, by TTE, LVEF was 54% (50.7%-55.5%), and by CMR, LVEF was 46% (39%-52.7%), mass 54.7 (48.5-59.5) g/m^2^, RVEF was 59% (52%-61.5%), and the total late gadolinium enhancement extent was 27.9 (18.4-39.0)%LV. The V̇O_2_ was 22.4 (18.7-24) mL/kg/min and 25% of patients presented an adverse remodeling.

### Reproducibility of Angio-IMR

The intraobserver reproducibility of Angio-IMR was excellent, with an ICC of 0.92 (95% CI, 0.80-0.96; *P* = .0001). Interobserver reproducibility was good, with an ICC of 0.81 (95% CI, 0.51-0.92; *P* = .0001).

## Discussion

In this pilot study, in patients with anterior STEMI, SSO_2_ improves CMD measured by Angio-IMR. Patient with high Angio-IMR at baseline (>40) presented more significant decrease.

In this study, CMD was assessed exclusively using Angio-IMR. No wire-based thermodilution IMR measurements were performed. These findings indicate that SSO_2_ was associated with microvascular improvement in the overall cohort, with a gradient of benefit favoring patients with higher baseline microvascular resistance. This pattern is consistent with a stratified medicine approach in which Angio-IMR helps identify those most likely to benefit, while not excluding potential improvement in others. This aligns with a stratified medicine approach, in which treatment intensity is tailored to microvascular status. In this context, Angio-IMR acts as a theragnostic biomarker, identifying patients with severe CMD who are most likely to benefit from adjunctive therapies such as SSO_2_. A theragnostic biomarker is a metric that predicts therapeutic response. The potential role of IMR or Angio-IMR could be that of a theragnostic biomarker to guide stratified medicine in STEMI patients.^10^ The development of stratified medicine is important for several reasons in these environments. The use of SSO_2_ equipment requires the patient to remain on the interventional cardiology table for 60 minutes, which may limit accessibility for other patients in centers with a single interventional cardiology room. Reducing the duration of the SSO_2_ procedure would be valuable but requires a full revalidation cycle of the method to ensure reproducibility and safety. A more promising approach would be to deliver the therapy outside the cath lab table, notably by securing catheter position during patient mobilization. Such adaptations could facilitate workflow integration and expand accessibility, especially in high-volume centers. In addition, SSO_2_ therapy has a cost and requires a second arterial puncture in the femoral artery, with a possible risk associated with this second puncture. It is, therefore, very interesting to reserve SSO_2_ for patients who will benefit most from it. The action on CMD monitored by IMR angiography may be considered a solution.

The use of a pressure/thermodilution wire during STEMI is clinically possible but presents several well-recognized drawbacks, notably, issues of trackability and hydrophobic coating in this acute setting. The possibility of angiography-derived IMR is very attractive. The development of an angiography-derived index by using computational fluid dynamics continues in several applications.^27^

These prospective results are in line with retrospective data using another commercial software (FlashAngio, Rainmed Ltd) based on the analysis of previous data studies using SSO_2_.^20^ In the overall population (ie, all patients included in the cohort, regardless of baseline microvascular status), there was no significant difference in Angio-IMR values before and after SSO_2_ (*P* = .21). However, SSO_2_ significantly reduced Angio-IMR in patients with CMD (defined as pre-SSO_2_ Angio-IMR >40), (pre-SSO_2_: 59.6 [IQR, 44.8-72.8] vs post-SSO_2_: 46.5 [IQR, 39.2-69.3]; *P* = .004) whereas no significant change was observed in patients without CMD (pre-SSO_2_ Angio-IMR ≤40) (32.2 [IQR, 26.9-36.6] vs 34.7 [IQR, 30.7-53.6]; *P* = .09).^28^ This difference with our results may be explained by the non-European origin of the cohort and, importantly, by the use of a different computational index in that study. Although the FlashAngio software also uses the term ‘Angio-IMR,’ its metric is derived from a proprietary algorithm that differs from the validated Angio-IMR methodology applied in the present study. A threshold of IMR >40 has been associated with worse prognosis, whether measured invasively or by Angio-derived IMR.^11^^,^^29^ In our study, we specifically used the validated Angio-IMR implementation, for which our group has previously demonstrated prognostic relevance.^24^

The main physiological action of SSO_2_ is to reopen the microcirculation by restoring endothelial function post-SSO_2_ therapy, as indicated in animal models.^12^^,^^18^ We can assume that SSO_2_ is only active in patients with CMD. Our data points in this direction, and these initial results are encouraging. These results need to be confirmed with more data, and thus, Angio-IMR could help to develop stratified medicine to use SSO_2_ in anterior STEMI patients with severe CMD. In contrast to systemic oxygen supplementation in normoxemic patients with acute MI, which has not demonstrated clinical benefit and has raised concerns regarding potential oxidative stress (eg, DETO2X-AMI and the SOCCER program^30^^,^^31^), SSO_2_ therapy delivers localized, high dissolved oxygen content directly into the infarct-related artery for a short, time-limited period during early reperfusion. This targeted intracoronary approach is designed to improve microvascular function without inducing prolonged systemic hyperoxia. In our cohort, no safety signals suggesting oxidative injury were observed, including hemodynamic instability, arrhythmias, or no-reflow, supporting the tolerability of localized SSO_2_ exposure. Nonetheless, these findings remain exploratory and warrant confirmation in larger controlled studies. Moreover, the correlation between IMR and angiography-derived IMR technology remains a matter of debate.^32^ In our setting, because each patient served as their own control, these debates were of lesser relevance.

We have chosen a single-arm study based on the literature on the damage to the microcirculation assessed by invasive IMR. The correlation between Angio-IMR and guide IMR has been demonstrated. At the time of PPCI, Angio-IMR and IMR exhibited significant correlation (*r* = 0.70, *P <* .001).^25^ Cuculi et al^33^ evaluated early changes in invasive measures of microvascular function by invasive IMR measures after PCI and 1 day before. After PPCI, median (IQR) IMR was 30 (18-65) in the low EF group and at day 1, median (IQR) IMR was 23 (15-40) (*P* = .64). In the high EF group, IMR was 37 (19-48) after PPCI, and 23 (18-34) at day 1 (*P* = .003). Patients with a low ejection fraction, as in our study, do not show a decrease in their invasive IMR measures over the first 24 hours without intervention. This suggests that the behavior of Angio-IMR is comparable to that of invasive IMR. In the present study, only Angio-IMR was assessed; mentions of wire-based IMR refer to supporting physiological evidence from prior studies.

In an animal study in a swine model of anterior MI by 90-minute balloon-inflation, the SSO_2_ effect on CMD was analyzed. Following 15-minute auto-reperfusion, animals underwent 120 minutes of intracoronary SSO_2_ infusion or standard reperfusion. Angio-IMR was measured at different time points, and regional MBF was evaluated by injection of microspheres.^34^ Overall, the median Angio-IMR values were not significantly different between the 2 groups at the end of the 90-minute balloon-occlusion (SSO_2_, 1.08 [0.76-1.30] vs control: 1.68 [1.05-2.85]; median [first and third quartiles], *P* = .15) and 105-minute timepoints (SSO_2_: 1.19 [0.96-1.26] vs control: 1.46 [1.17-1.72]; *P* = .12). At the 225-minute timepoint (end of SSO_2_ infusion), the median Angio-IMR value for the SSO_2_ therapy group was significantly lower than the corresponding baseline median Angio-IMR value (0.87 [0.85-0.94] vs 1.0 [1.0-1.0]; *P* = .024). In contrast, at the same timepoint, the median Angio-IMR value for the control group was significantly higher than the baseline median Angio-IMR value (1.69 [1.24-1.95] vs 1.0 [1.0-1.0]; *P* = .024). Moreover, we saw improvements in myocardial blood flow for 45 minutes after removal of the occlusion. It was only after that time that reperfusion injury started to set in, where there was a significant decrease in blood flow. These results point to the dynamic and progressive nature of microvascular dysfunction that has not been adequately described in humans.^35^ On the other hand, our results may be implied by the statistical phenomenon of regression to the mean, where extreme results, in this case, very high Angio-IMR, tend to return to the mean in subsequent evaluations. For this reason, we have no data available in humans on the spontaneous evolution of Angio-IMR, and hence, a randomized study with baseline and 60-minute Angio-IMR measurements in a control group is required, and these results need to be confirmed with more data. As no existing clinical data sets include serial Angio-IMR measurements performed both immediately post-PCI and again 60 minutes later under controlled hemodynamic conditions, matched retrospective comparisons are not feasible. The present pilot study has led to the development of a prospective randomized controlled trial in which Angio-IMR will be assessed before and after the 60-minute period in both the SSO_2_ and control groups, thereby enabling a formal comparison of microvascular effects.

### Limitations

This is a single-center proof-of-concept study. The results need to be confirmed by a larger study and randomized. The reliability of the measurement during the procedure must be carefully assessed. In our study, the results were satisfactory, but the measurements were performed away from the revascularization procedure by highly experienced operators. Extending angiography-derived IMR measurement to an entire team for routine clinical practice, particularly in emergency settings, may be more challenging, although automation of the measurement process is becoming increasingly available. Recent advances in semiautomated Angio-IMR software now allow in-lab computation within a few minutes by trained cath lab personnel, making real-time stratification feasible in the routine workflow. In this context, a potential therapeutic strategy could be proposed, whereby culprit LAD stenting is first performed, followed by immediate post-PCI Angio-IMR assessment while the patient remains on the catheterization table, allowing real-time identification of patients with significant residual microvascular dysfunction who may be candidates for adjunctive intracoronary SSO_2_ therapy. This concept, although not formally tested in the present study, provides a rationale for future Angio-IMR-guided trials evaluating targeted microvascular therapies.

## Conclusion

In patients with anterior STEMI, SSO_2_ therapy improved CMD as assessed by Angio-IMR, with the greatest benefit observed in patients presenting with elevated baseline Angio-IMR (>40). These preliminary results support the feasibility of using Angio-IMR during primary PCI to identify patients who may derive the most benefit from SSO_2_ therapy, a finding that warrants confirmation in larger cohorts.
